# Enabling automated herbarium sheet image post‐processing using neural network models for color reference chart detection

**DOI:** 10.1002/aps3.11331

**Published:** 2020-03-02

**Authors:** Dakila A. Ledesma, Caleb A. Powell, Joey Shaw, Hong Qin

**Affiliations:** ^1^ Department of Computer Science and Engineering University of Tennessee at Chattanooga Chattanooga Tennessee USA; ^2^ Department of Biology, Geology and Environmental Science University of Tennessee at Chattanooga Chattanooga Tennessee USA

**Keywords:** automation, digitization, herbarium, machine learning, natural history collections, specimen images

## Abstract

**Premise:**

Large‐scale efforts to digitize herbaria have resulted in more than 18 million publicly available Plantae images on sites such as iDigBio. The automation of image post‐processing will lead to time savings in the digitization of biological specimens, as well as improvements in data quality. Here, new and modified neural network methodologies were developed to automatically detect color reference charts (CRC), enabling the future automation of various post‐processing tasks.

**Methods and Results:**

We used 1000 herbarium specimen images from 52 herbaria to test our novel neural network model, ColorNet, which was developed to identify CRCs smaller than 4 cm^2^, resulting in a 30% increase in accuracy over the performance of other state‐of‐the‐art models such as Faster R‐CNN. For larger CRCs, we propose modifications to Faster R‐CNN to increase inference speed.

**Conclusions:**

Our proposed neural networks detect a range of CRCs, which may enable the automation of post‐processing tasks found in herbarium digitization workflows, such as image orientation or white balance correction.

Efforts to digitize biological collections have produced millions of herbarium specimen records and associated images, which are shared through websites such as iDigBio (https://www.idigbio.org) and the Southeast Regional Network of Expertise and Collections portal (SERNEC, http://sernecportal.org/portal/). Despite the large volume of specimen images and the prior use of artificial intelligence in herbaria for tasks such as phenological scoring (Lorieul et al., 2019), very few applications have been developed to automate the tasks involved in image post‐processing (i.e., preparing archival images for public access and use). Recommendations for tools, as well as step‐by‐step guidelines on how to use them for image capture and manual batch image post‐processing tasks, are available on iDigBio's website (Nelson et al., 2012; Tulig et al., 2012). Among the equipment recommended for imaging is a standardized color reference chart (CRC), which may be used later for color normalization during post‐processing (Nelson et al., 2015). Currently, post‐processing tasks using CRCs require manual intervention by trained technicians, proprietary software, as well as time for the import and export processes, requiring considerable amounts of time for technician training and their processing of images. As such, it has become increasingly relevant to automate these tasks, which will decrease the time and effort necessary for preparing specimens for upload, both by allowing for image post‐processing in real time as the images are captured and by removing the assumption/requirement that all images within a batch were taken with the same lighting conditions. Automation may therefore expedite the upload and availability of digitized specimens to the myriad of user groups, including researchers, educators, students, conservationists, enthusiasts, and the general public. Here, we present the use of machine learning to identify the CRC within a specimen image, along with supplemental proof‐of‐concept white balance and image rotation post‐processing code, as a first step to automating these post‐processing tasks.

In recent years, many advancements in computational power, computer vision (computer processing of image data), and machine learning have allowed for the accurate detection of objects within an image. This advancement is commonly attributed to the creation of modern convolutional neural networks (CNN) and region proposal algorithms/networks (LeCun et al., 2015). When a region proposal network (responsible for determining *where* objects of interest are located within a larger image) and a CNN classifier (responsible for identifying *what* the object is) are combined, they are typically called a regional convolutional neural network (R‐CNN). R‐CNNs are currently the most common and performant category of neural network for general image‐based object detection tasks (LeCun et al., 2015). However, even the most sophisticated R‐CNNs exhibit problems detecting objects when they are small relative to the image size or resolution, as is the case with the small CRCs commonly found in digitized herbarium specimens hosted on SERNEC (Fig. 1A). These small, hard‐to‐detect CRCs are defined in this paper as those smaller than 4 cm^2^ (e.g., the Image Science Associates ColorGauge Nano Target [Image Science Associates, Williamson, New York, USA]). In contrast, large CRCs found in digitized specimens hosted on SERNEC, which have sizes ranging from 10‐fold (CameraTrax 24ColorCard‐2x3 [https://www.cameratrax.com/]) to more than 19‐fold (Kodak Q‐13 [Kodak, Rochester, New York, USA]) larger than the small CRCs, pose no detection problems for current R‐CNN architectures. Much of the detection problems when using small CRCs are a result of the high‐resolution images required for archival specimen image data, which quadratically increases the amount of computation time and memory needed (Pavel and David, 2013). The resolution of these images is usually 3000 × 4000 pixels (px) or higher, in contrast to the current convention for image resolution of roughly 600 px on the shortest side of the image for R‐CNNs such as Microsoft's Faster R‐CNN (Ren et al., 2015). Processing herbarium specimens at the original resolution is therefore impossible, but downscaling the image resolution may cause much of the small CRC information to be lost during processing and may cause difficulty in detection (Fig. 1B, C). In addition, neural network prediction speed is commonly assessed using a graphics processing unit (GPU), with GPUs being 99× faster than a central processing unit (CPU) in neural network processing tasks (Lawrence et al., 2017). However, most herbarium digitization workstations do not possess a GPU properly supported by neural network libraries and may therefore be at an extreme speed deficit.

**Figure 1:**
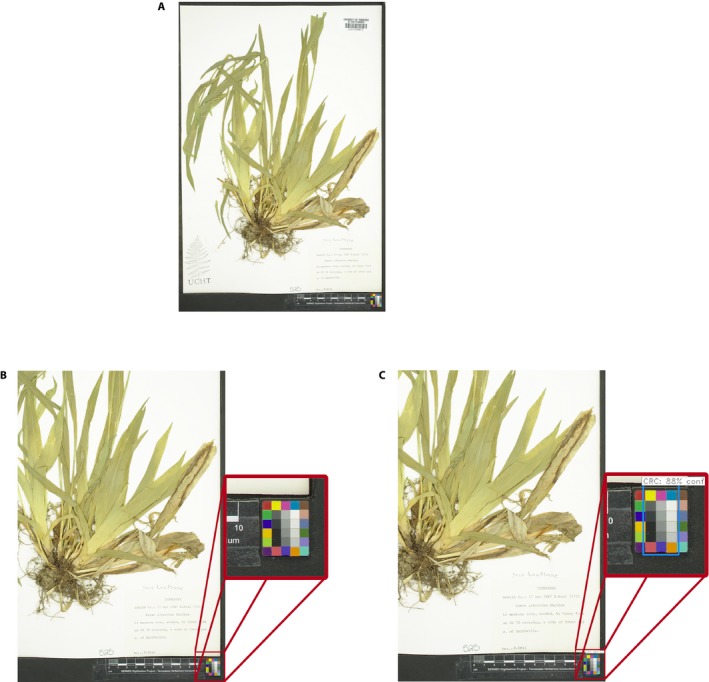
Example herbarium sheet image containing a small color reference chart (CRC) and its evaluation using Faster R‐CNN. (A) A herbarium sheet image with a small CRC showing the difference in scale between the CRC and the total image size. (B) The same image as in A*,* but with moderate cropping. Faster R‐CNN was not able to find the CRC even with this cropping due to its small relative size. (C) The same image as in A, but with more cropping than in B. Faster R‐CNN was able to find the CRC with 88% confidence, but the predicted region (blue box) lacks precision and would fail our standards for a correct CRC identification.

Thus, it was essential to create a new neural network architecture and modifications to current network architectures specifically designed to detect CRCs in herbarium sheets. To address this challenge, we established three design tenets: (1) the models must be able to accurately detect both small and large CRCs; (2) the models must be quick, allowing for real‐time detection while imaging herbarium sheets, so that real‐time verification by the operator may be performed and to prevent neural network inference from slowing the image capture process; and (3) the models must be able to run on equipment that is affordable and likely to be used across a wide variety of herbaria, and as such on computers without GPUs. Because of these criteria, we developed methodologies that were employed in our R‐CNN‐like network, ColorNet, as well as our modified Faster R‐CNN model. We compare these novel networks with the Selective Search region proposal algorithm (Uijlings et al., 2013)—a starting‐point region proposal algorithm for R‐CNNs such as the University of California, Berkeley's (UC Berkeley) R‐CNN model (Girshick et al., 2014), as well as an original, unmodified version of Microsoft's Faster R‐CNN—a representative of state‐of‐the‐art R‐CNNs that possesses one of the most robust region proposal networks (Ren et al., 2015).

## METHODS AND RESULTS

### Design methodology

Following the design goals mentioned above, the neural networks were specifically designed to be quick and accurate at detecting CRCs of various sizes, dimensions, and positions within herbarium sheet images. Current modern R‐CNN networks, for example, PASCAL VOC, COCO, and ImageNet (Deng et al., 2009; Everingham et al., 2010; Lin et al., 2014), are typically trained on general use‐case data sets containing several different types of objects (e.g., cars, animals, people); therefore, most, if not all, of the current state‐of‐the‐art R‐CNN networks were designed to be robust against the variety of different objects that need to be classified in these data sets (Girshick et al., 2014; Sandler et al., 2018; Howard et al., 2019). However, because our models only need to detect a CRC, we can forgo many of the complex techniques currently found in common state‐of‐the‐art neural networks and design domain‐specific neural networks that perform well on herbarium imaging stations.

### Processing and detection of small CRCs

#### Region proposal

**Figure 2:**
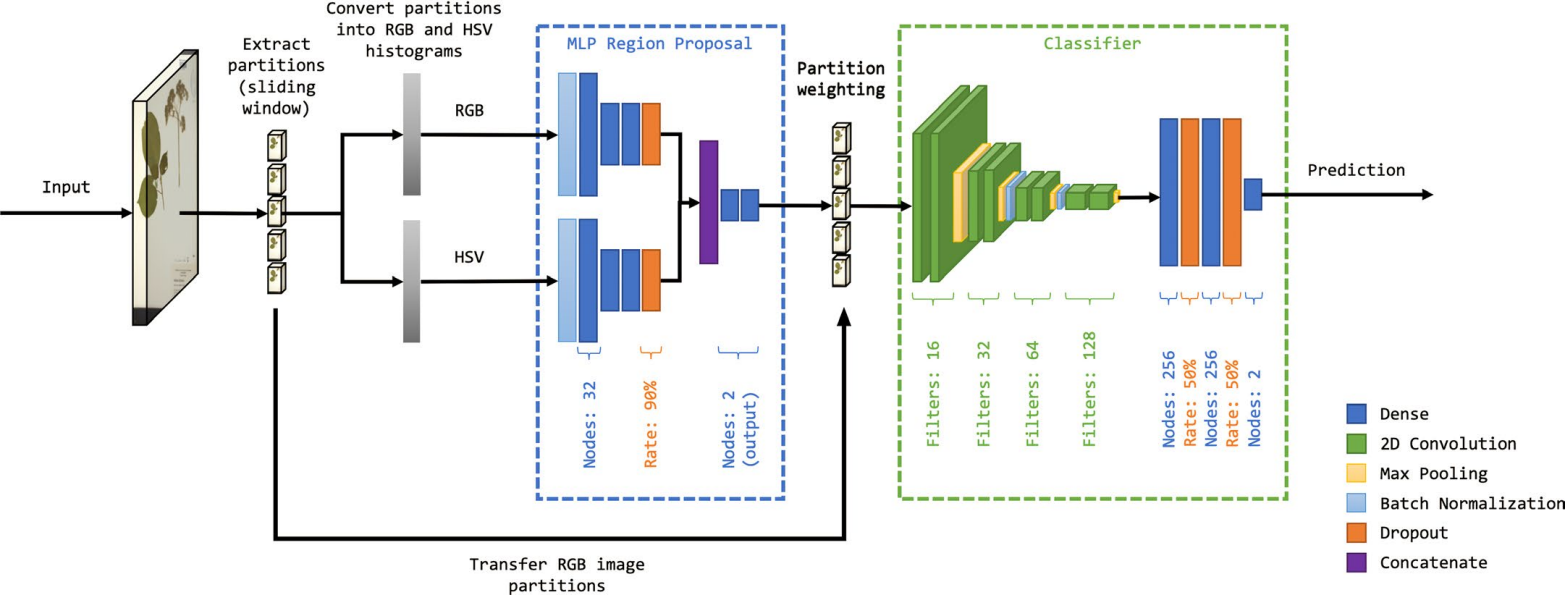
General ColorNet architecture. Specific details of each layer and their configuration may be found in Appendix S1. HSV: hue, saturation, value; MLP: multilayer perceptron; RGB: red, green, blue.

For the architecture (Fig. 2, Appendix S1) and design of ColorNet, the neural network created for the detection of small CRCs, two different methods were used to identify regions of interest: ColorNet‐Normal and ColorNet‐Quick. ColorNet‐Normal uses a sliding window technique that scans through the entire image, enabling the computation of relatively smaller areas of interest within the image. ColorNet‐Quick, on the other hand, uses OpenCV's (https://github.com/opencv/opencv) find squares method to detect squares that are appropriately sized for a small CRC. If appropriately sized squares are found, they are then used as partition proposals for the neural network. If no appropriate squares containing the CRC are found, ColorNet then falls back to using ColorNet‐Normal methods. Calculating only square‐like objects allows ColorNet‐Quick to be more than twice as fast as ColorNet‐Normal, but with a small loss in accuracy.

Unlike other neural networks, however, we do not compute the full RGB (red, green, blue) pixel information, which is three dimensional (width, height, RGB channels) for the inference of a particular region. Instead, we use color histograms, a one‐dimensional vector that contains the number of pixels representing a specific color. The use of color histograms and the associated reduction in dimensionality significantly decreases the number of calculations needed to be performed. In a 125 × 125 px image partition obtained from the sliding window, for example, 46,875 (125 × 125 × 3; 3 for red, green, and blue) variables would have to be processed. The number of variables in a color histogram does not vary with the size of the image; regardless of the image size, the vector size of a color histogram will always be 768 elements long for the RGB and HSV (hue, saturation, value) color space. Thus, in the 125 × 125 px image partition above, the use of color histograms would only amount to 768 variables to be inferred per color space, or, as in our case where both RGB and HSV color spaces were used, only 768 × 2 = 1536 variables had to be processed. Another advantage of having one‐dimensional data comes in the feasibility of using multilayer perceptron (MLP) networks (LeCun et al., 2015). Unlike CNNs, MLP networks do not attempt to infer spatial correlations, which are not present in one‐dimensional data, so MLP networks are less computationally expensive for processing these data. The dimensionality reduction through the use of color histograms and the speed increase from the use of MLP networks enables the processing of images at a much higher resolution (1250 × 1875 px) than the conventional R‐CNN processing resolutions (e.g., an equivalent resolution of 600 × 900 px for Faster R‐CNN). Processing the images at 1250 × 1875 px, while still smaller than the original digitized specimen resolution, was chosen for speed and better CPU computational feasibility without losing too much information for prediction accuracy. We chose 125 × 125 px as the size of the sliding window, which was large enough to capture small CRCs, and 25 px as the stride length (i.e., the number of pixels the sliding window moves horizontally and vertically).

#### Classification

We found that the processing of color histograms alone may be insufficient to identify a CRC in some cases, such as when the CRC is partially obstructed. The obfuscation of color information may lead other regions of the image to be more likely to be a CRC, especially in areas containing a high number of colors (e.g., flowers or colorful logos). Therefore, in order to circumvent problems that may arise when relying solely on color histograms, in the same vein as other R‐CNNs, a CNN classifier for determining the type of object (background or CRC) was used after the regions of interest were determined. In order to maintain the design principle of speed, we used our MLP proposal network to rank the partitions from the most probable to be a CRC to the least probable. This would allow our relatively computationally expensive CNN classifier (which processes three‐dimensional data) to process partitions starting from the most probable partition, reducing the amount of partitions processed down to ~5 partitions instead of computing the full ~3160. The average number of partitions processed is also much lower than the amount processed using Faster R‐CNN, in which 300 to 2000 proposals are typically processed by its CNN classifier.

**Figure 3:**
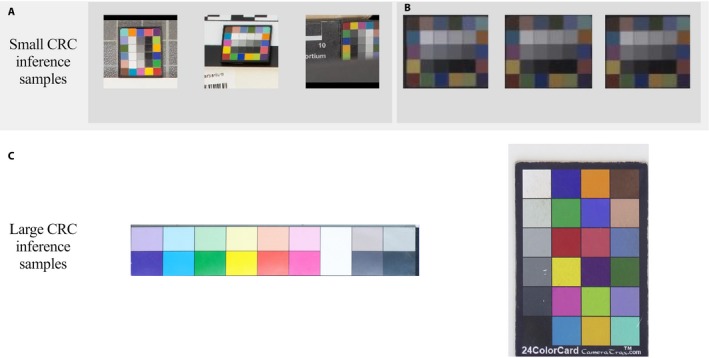
Example CRC detection results (not to scale) using ColorNet (small CRCs) (A, B) and modified Faster R‐CNN (large CRCs) (C), and post‐processing high‐precision crop results using the CRC information (B). Examples of small CRC detection: (A) The output of the most probable partition to contain the CRC. From left to right are regular, skewed, and partially obstructed small CRCs found within our test data set. (B) Outputs similar to A, but with high‐precision cropping of RAW images that have not been white balanced or color corrected.

Lastly, an optional high‐precision cropper is included to automatically perform the post‐processing of the predicted CRC region. The neural network pair is only able to identify the *partition* within the image that contains the CRC; thus, further cropping of the CRC using this high‐precision cropper would allow for more accurate color representation when being used to automate the white balance or color correction. The results of the high‐precision cropping may be seen in Fig. 3B.

### Processing and detection of large CRCs

The large CRCs are big enough to retain sufficient information for detection after the herbarium images are downsized to 600 × 900 px, meaning they may be adequately identified using R‐CNNs such as Faster R‐CNN. We opted to modify Faster R‐CNN to significantly reduce the neural network hyperparameters for the nodes and filters within various parts of the model (the specific changes may be found in Appendix 1). These changes resulted in a reduction of total neural network parameters from the normal 136,688,504 down to 8,558,600. We made these reductions to increase the inference speed of the neural network and decrease the computational power required, as the originally high number of network parameters (nodes/filters) were employed to properly assess morphic shapes (e.g., people, animals), which was unnecessary due to the non‐morphic shape of CRCs. The modified model still maintained very high region proposal precision and accuracy during the prediction of large CRCs.

### Training and testing data set

We trained the models on a training data set consisting of 3344 images from 164 collections found on the SERNEC Data Portal (collections may be found in the Literature Cited and Appendix S2), which was augmented to account for different lighting and camera conditions during the image capture of a specimen. The augmentations included laying CRCs on top of other parts of the image, darkening, desaturation, white balance shifting, and the rotation of images, leading to ~3.5 million 125 × 125 px JPG region/partition training samples for ColorNet and ~700,000 600 × 900 px equivalent JPG training samples for all Faster R‐CNN models (Selective Search is an algorithm and does not need training). To overcome the small CRC accuracy challenges encountered when using Faster R‐CNN, additional training data sets were explored using multiple image pre‐processing techniques to improve accuracy while still maintaining speed. For example, we tried partition sizes of 1250 × 150 px or 1875 × 150 px for training and testing, as well as laying these partitions onto a square crop filled with black (0 values) to circumvent the arbitrary resizing of Faster R‐CNN. Unfortunately, neither method improved prediction accuracy.

The testing data set comprised a total of 988 randomly pulled images not found within our training data set. These images were sourced from every herbarium collection within the SERNEC consortium that had a Darwin Core archive readily available, resulting in specimens from 58 different herbaria (Appendix S2). Six of these collections were omitted because they contained fewer than 19 samples in their Darwin Core archive. Nineteen images were randomly pulled from the Darwin Core archive of each collection for a total of 988 images; 565 were images with a small CRC, 395 were images with a large CRC, and 28 were omitted due to having no CRC or a CRC without a pure white and pure black color reference patch. The testing data set included a black padding at the border of the images, as it improved the prediction accuracy of both the small and large CRCs when using Faster R‐CNN. This improvement may arise from cross‐boundary anchor predictions (predictions outside the image bounds) being ignored during the training of the neural network, as cited in the original Faster R‐CNN paper (Ren et al., 2015). Within our testing data set, the CRCs fell into five types: Image Science Associates ColorGauge Nano, Kodak Q‐13, X‐Rite Colorchecker Passport (X‐Rite, Grand Rapids, Michigan, USA), X‐Rite Colorchecker Classic, and the CameraTrax 24ColorCard‐2x3.

### Results

The ColorNet and modified Faster R‐CNN models both resulted in an accuracy increase of up to 30.97%, along with a 2–8× reduction in computation time when tested against the Selective Search algorithm and the original Faster R‐CNN model, as seen in Table 1. The Selective Search algorithm was chosen for comparison as it is a region proposal algorithm that serves as a basis for R‐CNN models such as UC Berkeley's R‐CNN (Girshick et al., 2014). Specifically, we use OpenCV's Python implementation of the Selective Search algorithm, using the “fast” preset with multithreading and built‐in optimization. In addition, we also compared our work with a Keras implementation of Microsoft's Faster R‐CNN (Ren et al., 2015), which we believe contains one of the most robust region proposal networks currently available. The accuracy of all models is human‐verified, with correct detection denoted as cropping at least 80% of the CRC itself with the whitest and blackest part of the CRC visible.

**Table 1:** Region proposal results from each algorithm and model based on the randomly pulled 565 small CRC and 395 large CRC images from SERNEC.

Algorithm/model	Image resolution (pixels)	Accuracy (%)	Intel Core‐i3 inference time per image (seconds)^a^	Raspberry Pi 4 inference time per image (seconds)
Selective Search (Fast)	1250 × 1875	100^c^	39.90^c^	Not tested
Original Faster R‐CNN (small CRC)	600 × 900^b^	67.080	4.02	61.42
Original Faster R‐CNN (large CRC)	600 × 900^b^	99.748	3.71	59.15
ColorNet‐Quick (small CRC)	1250 × 1875	95.221	0.59	2.88
ColorNet‐Normal (small CRC)	1250 × 1875	98.053	1.11	4.68
Modified Faster R‐CNN (large CRC)	600 × 900^b^	99.241	1.45	7.08

a From the start of the inference time to the end of inference time, not including load times of the images or processing of the image.

b With the neural network resizing to proper dimensions (600 px on the shortest side) resulting in the processing of 600 × 900 px images.

c Region proposal only. No classifier was used due to very poor speed. The poor speed would be compounded with a classifier assessing each individual region for a CRC.

All performance results were derived using CPUs: an Intel Core i3‐4150 CPU (Intel, Santa Clara, California, USA), which we believe resembles a “typical” baseline CPU for a herbarium imaging computer, and a Raspberry Pi 4 (Raspberry Pi Foundation, Cambridge, United Kingdom), which could be a good low‐cost alternative for running the neural networks and automating the processing of herbarium sheet images. In terms of performance, our solutions provide the most benefit for Raspberry Pi 4 users, achieving more than an eight‐fold increase in inference time compared with Faster R‐CNN when run on the same device. During our experimentation, Selective Search required a significant amount of time to make the proper region proposals. The original Faster R‐CNN model, on the other hand, had difficulties properly assessing small CRCs when processing images with its arbitrary resizing down to 600 × 900 px. In addition, even with the reduced dimensionality, original Faster R‐CNN is still roughly three‐fold slower than our proposed neural network during prediction when using a desktop/laptop CPU.

## CONCLUSIONS

ColorNet and Modified Faster R‐CNN are over 98% effective at finding CRCs on herbarium specimen images, which may set the stage for the automation of image‐processing tasks and subsequent metadata enhancement, such as improving the white balance or proper image orientation. As a proof of concept, we show in Appendix S3 the automatic correction of highly white balance–shifted, darkened, and wrongly rotated images using a max white algorithm processed on our identified CRC and rotated using the quadrant of the location where the CRC is found. This proof‐of‐concept code, model source codes, and the trained model files of both ColorNet and modified Faster R‐CNN are available on GitHub at https://github.com/bgq527/ColorNet under the MIT license, allowing for full modification and reuse with no copyleft restriction. Furthermore, these models can serve as a first step in the development of other digitization post‐processing‐related tools, such as HerbASAP, a larger digitization support program currently in development that will allow the easy integration of these tools for herbarium image post‐processing (available at https://github.com/CapPow/HerbASAP). In the future, advancements and modifications within ColorNet may allow it to detect other small objects, such as very small phenological details, that may pose the same detection problems found within current R‐CNNs.

## Supporting information


**APPENDIX S1.** Detailed ColorNet architecture.

**APPENDIX S2.** Publishers of SERNEC biodiversity data (accessed through the SERNEC Data Portal, http://sernecportal.org/portal/index.php, 30 August 2019).

**APPENDIX S3.** Proof‐of‐concept post‐processing code (Jupyter Notebook) and details.

## Data Availability

Herbarium specimen images were acquired from the SERNEC Data Portal (http://sernecportal.org/portal/). The neural network models as developed in this paper, as well as model source code and information on the herbarium specimens used (SERNEC occurrence ID, the Index Herbariorum acronym of the associated collection, the catalog number, and the image URL as provided by SERNEC), are available on GitHub (https://github.com/bgq527/ColorNet).

## Appendix 1 Changes from original to modified Faster R‐CNN.

The code for this modified Faster R‐CNN is also provided on GitHub (https://github.com/bgq527/ColorNet).

Changes to Faster R‐CNN hyperparameters are as follows:

- VGG Base Network
  ◦ Block 1: Conv2D Filters 64 → 16
  ◦ Block 2: Conv2D Filters 128 → 32
  ◦ Block 3: Conv2D Filters 256 → 64
  ◦ Block 4: Conv2D Filters 512 → 128
  ◦ Block 5: Conv2D Filters 512 → 128
- Region Proposal Network
  ◦ 1st Conv2D: Filters 512 → 128
- Classifier
  ◦ Input_shape: (# of RoIs, 7, 7, 512) → (# of RoIs, 7, 7, 128)

Time Distributed Dense: Nodes 4096 → 1024
