# Supplementary material for: Enabling automated herbarium sheet image post‐processing using neural network models for color reference chart detection
Source: Appl Plant Sci. 2020 Mar 2;8(3):e11331. doi: 10.1002/aps3.11331 (PMC7073326; doi:10.1002/aps3.11331)
Supplement: Supplementary file 1 — APPENDIX S1. Detailed ColorNet architecture. [file APS3-8-e11331-s001.docx]

**APPENDIX S1.** Detailed ColorNet architecture.

| Image input | | | |
| --- | --- | --- | --- |
| Image RGB histogram | | Image HSV histogram | |
| Batch normalization | | Batch normalization | |
| Dense | Nodes: 32  Activation: tanh  Kernel regularizer: L2(0.001)  Kernel initializer: He uniform* | Dense | Nodes: 32  Activation: tanh  Kernel regularizer: L2(0.001)  Kernel initializer: He uniform* |
| Dense | Nodes: 16  Activation: tanh  Kernel regularizer: L2(0.01) | Dense | Nodes: 16  Activation: tanh  Kernel regularizer: L2(0.01) |
| Dropout | Rate: 90% | Dropout | Rate: 90% |
| Concatenate | | | |
| Dense | | Nodes: 8  Activation: tanh  Kernel regularizer: L2(0.001)  Activity regularizer: L2(0.001) | |
| Softmax Output (Dense) | | Nodes: 2  Activation: Softmax  Kernel regularizer: L2(0.001)  Activity regularizer: L2(0.001) | |
| Image partition probability and variance calculation (weighting) | | | |
| Image input (highest weights first) | | | |
| Conv2D | | Filters: 16  Filter Size: (3, 3)  Activation: ReLU | |
| Conv2D | | Filters: 16  Filter Size: (5,5)  Activation: ReLU | |
| MaxPool | | Size: (2, 2) | |
| Conv2D | | Filters: 32  Filter Size: (3, 3)  Activation: ReLU | |
| Conv2D | | Filters: 32  Filter Size: (5, 5)  Activation: ReLU | |
| MaxPool | | Size: (2, 2) | |
| Batch normalization | | | |
| Conv2D | | Filters: 64  Filter Size: (3, 3)  Activation: ReLU | |
| Conv2D | | Filters: 64  Filter Size: (5, 5)  Activation: ReLU | |
| MaxPool | | Size: (2, 2) | |
| Batch normalization | | | |
| Conv2D | | Filters: 128  Filter Size: (3, 3)  Activation: ReLU | |
| Conv2D | | Filters: 128  Filter Size: (5, 5)  Activation: ReLU | |
| MaxPool | | Size: (3, 3) | |
| Flatten | | | |
| Dense | | Nodes: 256  Kernel Regularizer: L2(0.001)  Activity Regularizer: L2(0.001)  Activation: ReLU | |
| Dropout | | Rate: 0.5 | |
| Dense | | Nodes: 256  Kernel Regularizer: L2(0.001)  Activity Regularizer: L2(0.001)  Activation: ReLU | |
| Dropout | | Rate: 0.5 | |
| Softmax output (dense) | | Nodes: 2  Activation: Softmax | |
| Image output | | | |
