# Supplementary material for: Enabling automated herbarium sheet image post‐processing using neural network models for color reference chart detection
Source: Appl Plant Sci. 2020 Mar 2;8(3):e11331. doi: 10.1002/aps3.11331 (PMC7073326; doi:10.1002/aps3.11331)
Supplement: Supplementary file 2 — APPENDIX S2. Publishers of SERNEC biodiversity data (accessed through the SERNEC Data Portal, http://sernecportal.org/portal/index.php, 30 August 2019). [file APS3-8-e11331-s002.docx]

**APPENDIX S2.** Publishers of SERNEC biodiversity data (accessed through the SERNEC Data Portal, http://sernecportal.org/portal/index.php, 30 August 2019).

Academy of Natural Sciences of Drexel University (PH); Adams State University Herbarium (ALAM); Albion College (ALBC); Appalachian State University, I. W. Carpenter, Jr. Herbarium (BOON); Arizona State University Vascular Plant Herbarium (ASU-Plants); Arizona Western College Herbarium (AWC); Arkansas Natural Heritage Commission Herbarium (ANHC); Arkansas State University (STAR); Arkansas Tech University Herbarium (APCR); Armstrong State University Herbarium (AASU); Austin Peay State University Herbarium (APSC); BRIT Philecology Herbarium (BRIT); Berea College, Ralph L. Thompson Herbarium (BEREA); Black Hills State University Herbarium (BHSC); Brown University Herbarium (BRU); Bureau of Land Management, Caliente Field Office (BLM-CFOBLM); Bureau of Land Management, Redding Field Office (BLM-RD); Burgundy Center for Wildlife Studies, Coopers Cove Herbarium (BCWS); Butler University, Friesner Herbarium (BUT); Calvin College Herbarium (CALVIN); Campbell University Herbarium (CAU); Canadian Museum of Nature (CMN-CANM); Carnegie Museum of Natural History Herbarium (CM); Carter Herbarium, Colorado College (COCO); Central Michigan University (CMC); Chicago Botanic Garden (CHIC); City of Alexandria Herbarium (AVCH); City of Boulder Herbarium (CIBO); Clemson University Herbarium (CLEMS); Colorado Mesa University, Walter A. Kelley Herbarium (MESA); Colorado State University Herbarium (CS); Converse College Herbarium (CONV); Delaware State University, Claude E. Phillips Herbarium (DOV); Delta State University (DSC); Denver Botanic Gardens, Kathryn Kalmbach Herbarium (DBG-KHD); Desert Botanical Garden Herbarium (DES); Duke University (DUKE); E. L. Reed Herbarium at Texas Tech University (TTC); East Carolina University Herbarium (ECUH); East Tennessee State University, John C. Warden Herbarium (ETSU); Eastern Kentucky University, Ronald L. Jones Herbarium (EKY); Eastern Michigan University Herbarium (EMC); Emory University Herbarium (GEO); Field Museum of Natural History (F-Botany); Florida State University’s Robert K. Godfrey Herbarium (FSU); Fort Lewis College Herbarium (FLD); Francis Marion University Herbarium (FMUH); Furman University, Ives Herbarium (FUGR); George Mason University, Ted R. Bradley Herbarium (GMUF-Plants); Georgia Southern University Herbarium (GAS); Georgia Southwestern State University Herbarium (GSW); Hendrix College Herbarium (HXC); Herbarium of the National Polytechnic Institute-Durango Unit (CIIDIR); Highlands Biological Station Herbarium (HBSH); Hillsdale College Herbarium (HLSD); Hope College Herbarium (HCHM); Howard University Herbarium (HUDC); Huntington University Herbarium (HUNT); Illinois Natural History Survey (ILLS); Indiana University Herbarium (Deam Herbarium) (IND); Institute for Botanical Exploration (IBE); Intermountain Herbarium (Utah State University) (USU-UTC); J. F. Bell Museum of Natural History Herbarium (MIN); James Madison University Herbarium (JMUH); Kent State University Herbarium (KE); Longwood University, Harvill-Stevens Herbarium (FARM); Louisiana State University Shreveport, D. T. MacRoberts Herbarium (LSUS); Louisiana State University, Shirley C. Tucker Herbarium (LSU-Vascular Plants); Lynchburg College, Ramsey-Freer Herbarium (LYN); Marie Selby Botanical Gardens Herbarium (SEL); Marshall University (MUHW); Maryland Department of Natural Resources (TAWES); Mecklenburg County Park and Recreation Herbarium (UNCC); Miami University, Willard Sherman Turrell Herbarium (MU); Michigan State University (MSC); Middle Tennessee State University (MTSU); Mississippi Museum of Natural Science Herbarium (MMNS); Mississippi State University (MISSA); Morehead State University Herbarium (MDKY); Morris Arboretum of University of Pennsylvania (MOAR); Morton Arboretum (MOR); Muhlenberg College (MCA); Museum of Northern Arizona (MNA); Natural History Institute Herbarium (NHI); Natural History Museum of Utah, Garrett Herbarium (UT-Botany); Navajo Nation Herbarium (NAVA); New York Botanical Garden, Steere Herbarium (NYBG); Newberry College Herbarium (NBYC); North Carolina Museum of Natural Sciences Herbarium (NCSM-NCSM); North Carolina State University Vascular Plant Herbarium (NCSC); North Carolina Zoological Park (NCZP); Northern Illinois University Herbarium (DEK); Northern Kentucky University, John W. Thieret Herbarium (KNK); Ohio State University Herbarium (OS); Ohio University, Bartley Herbarium (BHO); Patuxent Research Refuge (Laurel, Maryland, USA) (USFWS-PRR); Pennsylvania State University Herbarium (PAC); Pipe Spring National Monument (NPS-PISP); Rhodes College Herbarium (SWMT); Rocky Mountain Biological Laboratory (RMBL); Salem College (SC); San Juan College Herbarium (SJNM); Santa Cruz Island Reserve Herbarium (SCIR); Seney National Wildlife Refuge (SENEY); Sierra Pacific Industries-Forestry (SPIF); Silver Bluff Audubon Center Herbarium (SBAC); Southeastern Louisiana University, Glen N. Montz Herbarium (SELU); Southern Utah University (SUU); Staten Island Museum (SIM); Stoneville Weed Science Laboratory Herbarium (SWSL); Stover-Ebinger Herbarium (Eastern Illinois University) (EIU); Sul Ross University, A. Michael Powell Herbarium (SRSC); Swaner Preserve and EcoCenter (SWANER); Tennessee Technological University Herbarium (HTTU); Rutgers University, Chrysler Herbarium (CHRB); Tulane University Herbarium (NO); U.S. Forest Service Southwestern Region (USFS-TEUI); University of Alabama (UNA); University of Arkansas Herbarium (UARK); University of California-Berkeley, Sagehen Herbarium (SCFS); University of Central Arkansas Herbarium (UCAC); University of Central Florida Herbarium (FTU); University of Colorado Museum of Natural History Herbarium Vascular Plant Collection (COLO-V); University of Florida Herbarium (FLAS); University of Georgia Herbarium (GA); University of Illinois Herbarium (ILL); University of Louisiana at Monroe Herbarium (NLU); University of Mary Washington Herbarium (MWCF); University of Maryland, Norton-Brown Herbarium (MARY); University of Michigan Herbarium (MICH); University of Mississippi, Thomas M. Pullen Herbarium (MISS); University of Nevada Herbarium (RENO-V); University of New Mexico Herbarium (UNM-Vascular Plants); University of North Carolina Chapel Hill Herbarium (NCU); University of North Carolina Pembroke Herbarium (PEMB); University of Notre Dame, Greene/Nieuwland Herbarium (ND); University of Richmond Herbarium (URV); University of South Alabama Herbarium (USAM); University of South Carolina Salkehatchie Herbarium (SALK); University of South Carolina Upstate Herbarium (USCS); University of South Carolina, A. C. Moore Herbarium (USCH); University of South Florida Herbarium (USF); University of Southern Mississippi Herbarium (USMS); University of Tennessee Herbarium (TENN); University of Tennessee Martin Herbarium (UTM); University of Tennessee at Chattanooga Herbarium (UCHT); University of Vermont, Pringle Herbarium (VT); University of West Florida (UWFP); University of West Georgia Herbarium (WGC); University of Wisconsin-LaCrosse (UWL); University of Wisconsin-Madison, Wisconsin State Herbarium (WIS); University of Wisconsin-Stevens Point, Robert W. Freckmann Herbarium (UWSP); University of the South, Sewanee Herbarium (UOS); Valdosta State University Herbarium (VSC); Vanderbilt University Herbarium (VDB); Virginia Commonwealth University Herbarium (VCU); Virginia Polytechnic Institute and State University, Massey Herbarium (VPI); Wake Forest University (WFU); West Virginia University Herbarium (WVA); Western Carolina University Herbarium (WCUH); Western Michigan University (WMU); Western State Colorado University (WSC); Weymouth Woods Sandhills Nature Preserve Herbarium (WEWO); Winthrop University Herbarium (WINU)
