# Supplementary material for: Enabling automated herbarium sheet image post‐processing using neural network models for color reference chart detection
Source: Appl Plant Sci. 2020 Mar 2;8(3):e11331. doi: 10.1002/aps3.11331 (PMC7073326; doi:10.1002/aps3.11331)
Supplement: Supplementary file 3 — APPENDIX S3. Proof‐of‐concept post‐processing code (Jupyter Notebook) and details. [file APS3-8-e11331-s003.pdf]

Ledesma et al.—Applications in Plant Sciences 2020 8(3)—Data Supplement S3
DOI

APPENDIX S3. Proof-of-concept post-processing code (Jupyter Notebook) and details.

A .zip of the Jupyter Notebook and required scripts may be found in the releases of the ColorNet GitHub Repository: https://github.com/bgq527/ColorNet/releases

A requirements.txt is included in the .zip file, which contains the PyPi libraries that need to be installed to your Python environment before usage. These libraries may be installed through terminal/command prompt using:

```
pip install -r /path/to/file/requirements.txt
```

The proof-of-concept code uses the following simple algorithm for white balance and brightness correction. The actual code implementation of this algorithm is vectorized, but a for-loop implementation is shown for ease of interpretation:

Algorithm 1. White balance and brightness correction algorithm.

procedure white_balance_img (*img*, *white_r*, *white_g*, *white_b*):
    Transpose *img* matrix from rows, columns, channels to channels, rows, columns
    for every *value* in image red channel:
        value ← Minimum of (*value* × (255 / *white_r*) – 0.18) and 255
    for every value in image green channel:
        value ← Minimum of (*value* × (255 / *white_g*) – 0.18) and 255
    for every value in image blue channel:
        value ← Minimum of (*value* × (255 / *white_b*) – 0.18) and 255
    Transpose *img* matrix from channels, rows, columns to rows, columns, channels
    return *img*

Some sample results from the Jupyter notebook may be seen here:

Automated post-processing (proof-of-concept results)

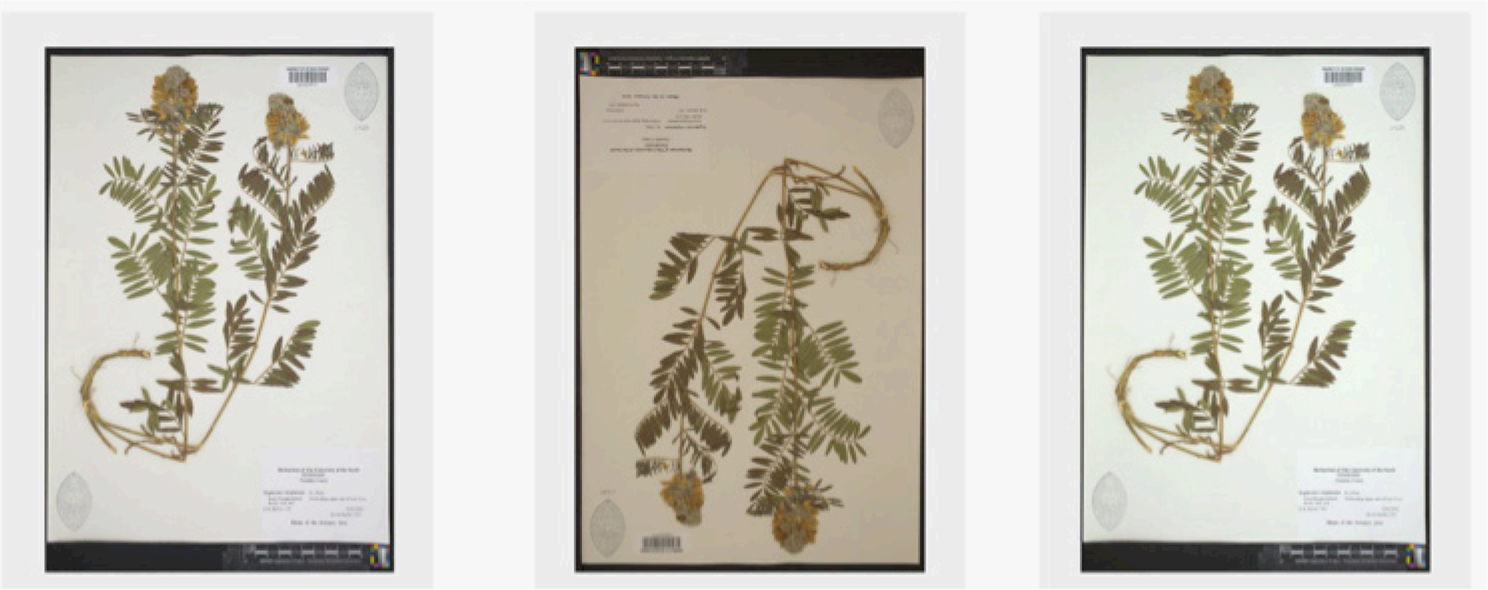

A **B** **C**

(A) A correctly post-processed image. (B) The same image from A, but white-balance shifted to 5200 K, brightness set to –50, and rotated 180° using the GNU Image Manipulation Program (https://www.gimp.org). (C) The reconstruction of B through the information found on the detected color reference charts (CRC) and a simple algorithm (found above). The algorithm uses CRC white patch for white balance and brightness correction. The image is rotated based on the CRC location's found quadrant and expected quadrant.
